# Identification of the role of oral health educators in elementary schools during COVID-19 pandemic: a competency framework

**DOI:** 10.1186/s13104-021-05887-z

**Published:** 2022-01-10

**Authors:** Adel Khiami, Mayssoon Dashash

**Affiliations:** 1 Demonstration Training and Research Oral Health Center (DTROHC), Ministry of Education, Alassad suburb, Damascus, Syria; 2 Medical Education Master Program, Syrian Virtual University, Damascus, Syria; 3 Pediatric Dentistry Department, Faculty of Dentistry, Damascus University, Damascus, Syria

**Keywords:** Oral health education, COVID-19, Educator, Competencies, Elementary school, Syria

## Abstract

**Objective:**

Oral health educators should have the required knowledge, skills and attitude in order to meet the increased needs of the Ministry of Education in Syria as well as to perform their duties in promoting oral health in children appropriately during the COVID-19 pandemic. Therefore, this study was undertaken to identify core competencies required for oral health educators in elementary schools during the COVID-19 pandemic. Qualitative exploratory study was undertaken. A focus group which consisted of 5 Medical Education postgraduates and 3 oral health educators’ training team members were invited to formulate a preliminary list of basic competencies. Delphi technique was also adopted through inviting 12 experts in oral health education to evaluate and formulate a final list of elementary schools’ oral health educators’ competencies during the COVID-19 pandemic.

**Results:**

A competency framework was developed. Fifty-five competencies were identified including 35 in cognitive domain, 12 skills and 8 attitude competencies. A list of essential competencies has been identified. These competencies should be addressed in training programs targeting oral health educators, which can consequently produce competent educators who can successfully promote and provide health care to all schoolchildren during the COVID-19 pandemic.

**Supplementary Information:**

The online version contains supplementary material available at 10.1186/s13104-021-05887-z.

## Introduction

Global health rapid changes related to Covid-19 pandemic [1], have enforced all health workers in all sectors to urgently call for developing competency framework for dealing with the pandemic [2–4].

Oral health educators during the COVID-19 pandemic have a critical role in fighting the pandemic in order to decrease the infection of children and the transmission to their family and society. In this regard, oral health educators in elementary schools should be able, during and after the COVID-19 pandemic [5, 6] to deliver health promotion and health care in elementary schools, and to establish healthy behavior in society [7, 8]. There is a need to design a competency framework that can guide decision makers to develop training programs that can enable oral health educators to provide health care appropriately.

The Demonstration, Training and Research Oral Health Center (DTROHC) in the Ministry of Education in the Syrian Arab Republic was established to provide different preventive, curative and educational programs [9] including training teachers and auxiliary teachers to introduce oral health education in schools [10] as Community Oral Health Workers (COHWs). However, there is no adopted systematic approach in the Centre in order to train oral health educators [11]. Unfortunately, the training is dependent on the experience of individuals who are responsible for the training process rather than the institution itself.

In fact, the situation during COVID-19 pandemic demands urgently more systematic approach.

In this regard, the competency based medical education (CBME) could have a vital role during the pandemic. It has attracted several policy-makers in health care professions in the World [12] with different methodologies adopted to define the core competencies [13]. For instance, Delphi technique, a consensus methodology that has been used extensively to define the competencies of health professionals, either alone [3], or in combination with focus group [14] or consultations meetings [15].

In this study, a competency framework was developed using Delphi and focus group methodologies to identify essential competencies of oral health educators of elementary schools’ during the COVID-19 pandemic.

## Main text

### Materials and methods

After obtaining the ethical approval from the ethical committee at the Syrian Virtual University SVU, two qualitative methods exploratory study design was undertaken. Two qualitative methodologies including the focus group and Delphi technique w ere sequentially applied.

For focus group, eight participants accepted to take part in the study in which 5 were postgraduate students in Medical Education program at the SVU (three graduated dentists, one graduated pharmacist and, one pulmonology specialist. and also three dentists who are employed in the DTROHC and work as oral health educators’ training team. The invitation was performed by the principal researcher directly or through phone. The focus group meeting was held in DTROHC. Full explanation about the research and strategy to be applied was given to focus group participants. Then, the DTROHC members were asked to give the focus group a brief report about the required tasks from the oral health educators in the elementary schools. This has led to identify the required competencies and to formulate a preliminary list of basic competencies for oral health educators in elementary schools. The meeting took two and a half hours.

Delphi technique was the second step of the process in identifying the essential competencies of oral health educators in elementary schools. Through three cycle of Delphi technique, the selected expert panel evaluated the initial competency list suggested by the focus group and suggested additional competencies in order to reach a consensus on a final core competency list for the oral health educators in the elementary schools.

Fifteen experts in oral health education included academic staff and experts in Pediatric Dentistry, Restorative Dentistry, Family Medicine and Public Health from Damascus University DU, Tishreen University TU, Syrian Private University SPU, Al Wataniya Private University AWPU, Syrian board family medicine expert, United Nations Relief and Works Agency UNRWA dental surgeon and DTROHC dentists were invited to take part in Delphis’ expert panel. 12 out of 15 accepted to participate. Communication with experts was performed using social media, or phone and Electronic informed consent was obtained.

Delphis’ questionnaires were developed sequentially for this study.

The 1st cycle Delphi questionnaire contained an explicit to the purpose of the questionnaire and the mechanism for preserving the confidentiality of the data and the exclusive use for research purposes only.

The questionnaire contained personal data fields, the initial list of competencies that were reached through the focus group categorized as (knowledge, skills and attitudes) and a field for suggestions provided by experts, the questionnaire adopted 4 point Likert scale.

The 2nd Delphi’s cycle questionnaire included an introduction which summarized the result of the first cycle while the questionnaire body contained the experts’ suggested competencies list for evaluation using the same way of Delphi’s First cycle while the 3rd Delphi’s cycle Questionnaire included the final list of competencies and a consensus question to be answered by the experts. All Delphi questionnaires were designed and implemented using the Google Forms web application. An English language version copy of the developed questionnaires can be found in Questionnaires Additional file 1.

The evaluation mean value of ≥ 2.5 has been considered acceptable for entering the next Delphi’s cycle.

In the 1st Delphi’s cycle, the experts evaluate the initial competency list suggested by the focus group according to their importance using a quad forced Likert scale [16] with the following codes: 1, 2, 3 and 4 corresponding to ‘not important’, ‘Slightly important’, ‘important’, and ‘essential’, respectively. In this cycle, experts also suggested additional competencies, which they found important. In the 2nd Delphi’s cycle the experts evaluated the additional suggested competencies in the same way used in 1st cycle.

In the 3rd Delphi’s cycle, a minimum level of consensus 80% is termed to accept the competency list as the final core competencies for oral health educators in elementary schools, where the level of consensus defined by the percentage of experts who agreed to the competencies final list.

The implementation of the Delphi technique took 50 days from 10/20/2020 until 10/12/2020.

Google Forms and the Statistical Package of Social Science software (IBM SPSS statistic version 26) were used for data collection and analysis. The responses were documented and presented with their corresponding values.

As percent agreement is one of the common way to define consensus in Delphi technique [3, 17], Descriptive statistics including median, Mean and standard deviation were calculated, consensus was defined as 62.5% agreement (or a mean score of 2.5) and above on each given statement in round 1 and 2 and 80% for overall final competency list agreement in the round 3.

### Results

At the end of the focus group meeting, a preliminary list of 49 competencies were identified. They were 31 Knowledge competencies arranged in 7 domains, 10 skills competencies arranged in 4 domains and 8 attitude competencies arranged in 3 domains.

The results of Delphi technique identified six additional competencies; 4 cognitive and 2 skills.

In the 2nd Delphi cycle, all 12 participating experts responded to the questionnaire, the additional suggested competencies were evaluated in the same way used in the first cycle.

**Fig. 1:**
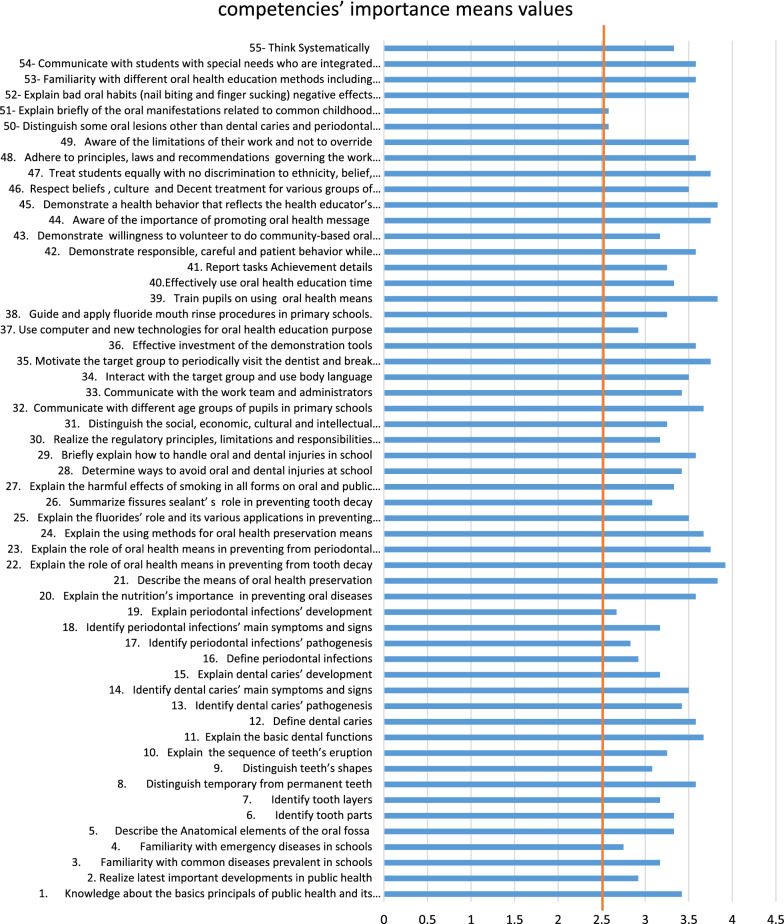
Competencies’ importance evaluation means values given by Delphi experts

All competencies’ evaluation mean values exceeded the termed threshold (≥ 2.5) as illustrated in Fig. 1.

the 3rd cycle was the final statement of group consensus; 11 of 12 experts responded to the questionnaire, the non-responding experts were considered not consensual with the list of competencies, all respondents to 3rd Delphi cycle approved consensus, which makes the consensus percentage equal to (91.66%), and since this percentage exceeds 80% (the termed minimum consensus rate in the present study), the proposed list of competencies can be considered as the final competencies’ list for oral health educators in elementary schools.

Table 1 shows the final competencies’ list for oral health educators in elementary schools in details.

**Table 1:** The final competencies’ list for oral health educators in elementary schools

**Cognitive (knowledge)**	
* Public health domain*	
Knowledge about the basics principals of public health and its maintenance including personal protection	
Realize latest important developments in public health	
Familiarity with common diseases prevalent in schools	
Familiarity with emergency diseases in schools	
Explain briefly the oral manifestations related to common childhood diseases	
* Description of the anatomical elements of the oral fossa domain*	
Describe the anatomical elements of the oral fossa	
*Describing and identifying the teeth’s anatomical elements domain*	
Identify tooth parts	
Identify tooth layers	
Distinguish temporary from permanent teeth	
Distinguish teeth’s shapes	
Explain the sequence of teeth’s eruption	
* Explanation of basic dental functions domain*	
Explain of basic dental functions	
*Oral and dental diseases domain*	
Define dental caries	Dental caries
Identify dental caries’ pathogenesis	
Identify dental caries’ main symptoms and signs	
Explain dental caries’ development	
Define periodontal infections	Periodontal infections
Identify periodontal infections’ pathogenesis	
Identify periodontal infections’ main symptoms and signs	
Explain periodontal infections’ development	
Distinguish some oral lesions other than dental caries and periodontal disease	Other
* The prevention of oral and dental diseases and injuries domain*	
Explain the nutrition’s importance in preventing oral diseases	
Describe the means of oral health preservation	
Explain the role of oral health means in preventing from tooth decay	
Explain the role of oral health means in preventing from periodontal infections	
Explain the using methods for oral health preservation means	
Explain the fluorides’ role and its various applications in preventing tooth decay	
Summarize fissures sealant’ s role in preventing tooth decay	
Explain the harmful effects of smoking in all forms on oral and public health	
Determine ways to avoid oral and dental injuries at school	
Briefly explain how to handle oral and dental injuries in school	
Explain bad oral habits (nail biting and finger sucking) negative effects on oral health	
* Other cognitive competencies domain*	
Realize the regulatory principles, limitations and responsibilities related to the health educators’ work in elementary schools	
Distinguish the social, economic, cultural and intellectual characteristics related to target groups	
Familiarity with different oral health education methods including online education [17]	
**Skills**	
* Communication skills domain*	
Communicate with different age groups of pupils in elementary schools	
Communicate with the work team and administrators	
Interact with the target group and use body language	
Motivate the target group to periodically visit the dentist and break the fear barrier	
Communicate with students with special needs who are integrated into schools	
* Tools investment domain*	
Effective investment of the demonstration tools	
Use computer and new technologies for oral health education purpose	
* Training domain*	
Guide and apply fluoride mouth rinse procedures in elementary schools	
Train pupils on using of oral health means	
*Organize domain*	
Effectively use oral health education time	
Report tasks achievement details	
Think systematically	
**Attitude**	
* Personal characteristics domain*	
Demonstrate responsible, careful and patient behavior while performing their work	
Demonstrate willingness to volunteer to do community-based oral health work	
Aware of promoting oral health message importance	
Demonstrate a health behavior that reflects the health educator’s image	
*Rights’ respect domain*	
Respect beliefs, culture and decent treatment for various groups of children, teachers, administrators and parents	
Treat students equally with no discrimination to ethnicity, belief, gender, or social background	
*Respecting laws and regulations domain*	
Adhere to principles, laws and recommendations governing the work of oral health educators to interact with children within elementary schools and to apply oral health education procedures and preventive measures	
Aware of the limitations of their work and not to override	

### Discussion

Several CBME frameworks were developed, with different competency structures [18]. Regardless of the type of framework that will be adopted, identifying competencies are the basic CBME elements [19].

Although globally developed frameworks [20] are of high importance, the differences driven by factors related to COVID-19 pandemic, country, legal, cultural and workplace specification should not be ignored. Consequently, specific strategy that can be used to define the required competencies for a group of health workers in the actual local settings must be adopted in order to meet local needs [21].

Kaufman and colleagues, have developed a list of competencies using focus group method. Members of focus group were stakeholders from parents, medical professionals, researchers and policy makers [22].

Patterson et al. have used job analysis methodology in order to define core and specific competencies using practice observation, stakeholders focus groups and interviews [23].

Several studies also have considered Delphi technique as a strategy of choice to define competencies. Batt and his colleagues, have indicated in their systematic review that 26% of included studies have used Delphi technique to define competencies in medicine and nursing [13].

Similar to other previous studies [3, 14, 15], the present study utilized two methodologies including focus group as a source to generate ideas for the initial bulk of competencies and the use of Delphi technique as a filter to reshape and complete the competency list as the strategy of choice.

Focus group method has been considered an effective and economical method for generating data in terms of time and cost [24] where the data collected are original and generated in a constructive manner and not according to pre-defined opinions [25].

In the present study, the focus group designed to include two types of participants. Firstly, postgraduates in medical education, for a set of specifications, including knowledge about competencies’ vocabulary and its’ formulation methods in addition to their knowledge and skills related to the specializations to which they belong to dentistry, medicine and pharmacology. Secondly, the focus group participants included all DTROHC oral health education training team. They were considered as stakeholders and expert dentists as they were involved in training process and were familiar with all required tasks of oral health education. The thing that gave them the ability to provide full description of educators’ work in a way that simulates the job analysis methodology used by Fiona Patterson et al. [23] in order to identify competencies.

The present study included experts from different institutions, medical schools, expertise and specialties. The criteria of selection included 5 years or more in the field of expertise or specialization in oral health education of children.

One attitude and five cognitive identified competencies were dedicated to public health which can be related to the augmented public health concerns due to COVID-19 pandemic [26].

The maximum value was for “*Explaining the role of oral health means in preventing from tooth decay*” competency as dental caries is still a major oral health problem for schoolchildren in Syria [27].

The Minimum value was for “*Distinguishing some oral lesions other than dental caries and periodontal disease*” which means that some experts believe that this competency is not essential for oral health educators should possess as many dentists themselves experience difficulties in daily practice in providing diagnosis to oral mucosal lesions [28].

Identifying competencies related to computer technology and online medical education has been considered important in keeping oral health educators up to date during the COVID-19 pandemic [29].

### Conclusion

A list of essential competencies has been identified, these competencies should be addressed in training programs targeting oral health educators, which will consequently produce competent educators who can successfully promote and provide health care.

## Limitations

Although being an important method, Delphi technique is time consuming method [30], as it took in the present study about 50 days to accomplish the Delphi process. Also limiting Delphi experts number to n = 12 caused by difficulties in widening experts list, risking experts low response rate, Which is one of the common Delphi’s limitation [31].

## Supplementary Information

**Additional file 1. **Delphi technique Questionnaires.

## Data Availability

The datasets generated and analyzed during the current study are available from the corresponding author on reasonable request, taking into consideration the adherence to ethical approval obtained for this study, and adherence to confidentiality.

## Abbreviations

DTROHC: Demonstration, training and research oral health center
COVID-19: Corona virus disease of 2019
COHWs: Community Oral Health Workers
CBME: Competency Based Medical Education
SVU: Syrian Virtual University
DU: Damascus University
TU: Tishreen University
SPU: Syrian Private University
AWPU: Al Wataniya Private University
UNRWA: United Nations Relief and Works Agency
