# Supplementary material for: Identification of the role of oral health educators in elementary schools during COVID-19 pandemic: a competency framework
Source: BMC Res Notes. 2022 Jan 10;15:6. doi: 10.1186/s13104-021-05887-z (PMC8744045; doi:10.1186/s13104-021-05887-z)
Supplement: Supplementary file 1 — Additional file 1. Delphi technique Questionnaires. [file 13104_2021_5887_MOESM1_ESM.docx]

**Additional file 1**

**Identification of the Role of Oral Health Educators in Elementary Schools during COVID-19 Pandemic: A Competency Framework.**

**Delphi technique Questionnaires**

**Introduction:**

Dear Expert, Within the procedures of a study titled “Identification of the Role of Oral Health Educators in Elementary Schools during COVID-19 Pandemic: A Competency Framework “aimed to identify the core competencies of oral health educators in primary schools in Syria during COVID-19 Pandemic, we hope to benefit as much as possible from your experience within your field of specialty and expertise through inviting you to participate in this study as Delphi technique experts list s’ member.

Participating in this research will play a role in guiding the training of competent oral health educators who can successfully promote and provide health care to all schoolchildren during the COVID-19 pandemic.

Participation in the research is voluntary and the experts who choose to participate in the study will be asked to assess the degree of importance of a group of competencies to be the core competencies of oral health educators in primary schools.

Delphi technique is one of the consensus consulting techniques.in this study evaluation will use 3 Delphi cycles:

In the 1st Delphi’s cycle, the experts will be asked to evaluate the initial competency list suggested by the focus group according to their importance as ‘not important’, ‘Slightly important’, ‘important’, or ‘essential’. In this cycle, Experts can also suggest additional competencies, which they found important.

In the 2^nd^ Delphi’s cycle the experts will be asked to evaluate the additional suggested competencies in the same way used in 1st cycle.

The 3^rd^ Delphi cycle will be the final statement of group consensus where the expert will Acknowledge their compliance or non-compliance regarding the developed list of competency.

Experts are free to participate or stop participating in the study in any time they chose.

Answering this questionnaire will be considered as an informed consent that authorizes us to use the data collected from your participation to be used for research purposes only. knowing that participants’ names will be acknowledged as Delphi experts in the acknowledgment section of the study.

To make your task easier, here is a small introduction explaining the work of the oral health educator in primary schools:

The oral health educator in the primary school is a teacher or auxiliary teacher who has received training courses in order to qualify him:

1- Providing oral health education in the classroom

2- Training the students on the implementation of the fluoride mouth rinses procedure and supervising its implementation

3- Training students to use oral hygiene methods

4- Providing advice and guidance related to maintaining public health

5- The oral health educator is not allowed to conduct any direct diagnostic, preventive or curative intervention on the target group members except for the application of the fluoride rinse procedure.

**Delphi s’ first cycle questionnaire**

**Expert name:** ______________________ **Email:** ________________________

**Specialization or expertise:** ___________________________________________________

**Affiliation:** _________________________________________________________________

Please Add ☑ in the box of the corresponding answer, which represents your evaluation about each competency to be a core competency for oral health educators in elementary schools in Syria during COVID- 19 pandemic.

**Cognitive(Knowledge)**

**public health domain**

1. Knowledge about the basics principals of public health and its maintenance including Personal protection.

🞎 not important 🞎 Slightly important 🞎 important 🞎 essential

1. Realize latest important developments in public health

🞎 not important 🞎 Slightly important 🞎 important 🞎 essential

1. Familiarity with common diseases prevalent in schools

🞎 not important 🞎 Slightly important 🞎 important 🞎 essential

1. Familiarity with emergency diseases in schools

🞎 not important 🞎 Slightly important 🞎 important 🞎 essential

**Description of the Anatomical elements of the oral fossa domain**

1. Describe the Anatomical elements of the oral fossa

🞎 not important 🞎 Slightly important 🞎 important 🞎 essential

**Describing and identifying the teeth’s anatomical elements domain**

1. Identify tooth parts

🞎 not important 🞎 Slightly important 🞎 important 🞎 essential

1. Identify tooth layers

🞎 not important 🞎 Slightly important 🞎 important 🞎 essential

1. Distinguish temporary from permanent teeth

🞎 not important 🞎 Slightly important 🞎 important 🞎 essential

1. Distinguish teeth’s shapes

🞎 not important 🞎 Slightly important 🞎 important 🞎 essential

1. Explain the sequence of teeth’s eruption

🞎 not important 🞎 Slightly important 🞎 important 🞎 essential

**Explanation of basic dental functions domain**

1. Explanation of basic dental functions

🞎 not important 🞎 Slightly important 🞎 important 🞎 essential

**Oral and dental diseases domain**

1. Define dental caries

🞎 not important 🞎 Slightly important 🞎 important 🞎 essential

1. Identify dental caries’ pathogenesis

🞎 not important 🞎 Slightly important 🞎 important 🞎 essential

1. Identify dental caries’ main symptoms and signs

🞎 not important 🞎 Slightly important 🞎 important 🞎 essential

1. Explain dental caries’ development

🞎 not important 🞎 Slightly important 🞎 important 🞎 essential

1. Define periodontal infections

🞎 not important 🞎 Slightly important 🞎 important 🞎 essential

1. Identify periodontal infections’ pathogenesis

🞎 not important 🞎 Slightly important 🞎 important 🞎 essential

1. Identify periodontal infections’ main symptoms and signs

🞎 not important 🞎 Slightly important 🞎 important 🞎 essential

1. Explain periodontal infections’ development

🞎 not important 🞎 Slightly important 🞎 important 🞎 essential

**The prevention of Oral and dental diseases and injuries domain**

1. Explain the nutrition’s importance in preventing oral diseases

🞎 not important 🞎 Slightly important 🞎 important 🞎 essential

1. Describe the means of oral health preservation

🞎 not important 🞎 Slightly important 🞎 important 🞎 essential

1. Explain the role of oral health means in preventing from tooth decay

🞎 not important 🞎 Slightly important 🞎 important 🞎 essential

1. Explain the role of oral health means in preventing from periodontal infections

🞎 not important 🞎 Slightly important 🞎 important 🞎 essential

1. Explain the using methods for oral health preservation means

🞎 not important 🞎 Slightly important 🞎 important 🞎 essential

1. Explain the fluorides’ role and its various applications in preventing tooth decay

🞎 not important 🞎 Slightly important 🞎 important 🞎 essential

1. Summarize fissures sealant’ s role in preventing tooth decay

🞎 not important 🞎 Slightly important 🞎 important 🞎 essential

1. Explain the harmful effects of smoking in all forms on oral and public health

🞎 not important 🞎 Slightly important 🞎 important 🞎 essential

1. Determine ways to avoid oral and dental injuries at school

🞎 not important 🞎 Slightly important 🞎 important 🞎 essential

1. Briefly explain how to handle oral and dental injuries in school

🞎 not important 🞎 Slightly important 🞎 important 🞎 essential

**Other cognitive competencies domain**

1. Realize the regulatory principles, limitations and responsibilities related to the health educators’ work in elementary schools

🞎 not important 🞎 Slightly important 🞎 important 🞎 essential

1. Distinguish the social, economic, cultural and intellectual characteristics related to target groups

🞎 not important 🞎 Slightly important 🞎 important 🞎 essential

**Skills**

**Communication skills domain**

1. Communicate with different age groups of pupils in elementary schools

🞎 not important 🞎 Slightly important 🞎 important 🞎 essential

1. Communicate with the work team and administrators

🞎 not important 🞎 Slightly important 🞎 important 🞎 essential

1. Interact with the target group and use body language

🞎 not important 🞎 Slightly important 🞎 important 🞎 essential

1. Motivate the target group to periodically visit the dentist and break the fear barrier.

🞎 not important 🞎 Slightly important 🞎 important 🞎 essential

**Tools investment domain**

1. Effective investment of the demonstration tools

🞎 not important 🞎 Slightly important 🞎 important 🞎 essential

1. Use computer and new technologies for oral health education purpose

🞎 not important 🞎 Slightly important 🞎 important 🞎 essential

**Training domain**

1. Guide and apply fluoride mouth rinse procedures in elementary schools.

🞎 not important 🞎 Slightly important 🞎 important 🞎 essential

1. Train pupils on using of oral health means

🞎 not important 🞎 Slightly important 🞎 important 🞎 essential

**Organize domain**

1. Effectively use oral health education time

🞎 not important 🞎 Slightly important 🞎 important 🞎 essential

1. Report tasks Achievement details

🞎 not important 🞎 Slightly important 🞎 important 🞎 essential

**Attitude**

**Personal characteristics domain**

1. Demonstrate responsible, careful and patient behavior while performing their work

🞎 not important 🞎 Slightly important 🞎 important 🞎 essential

1. Demonstrate willingness to volunteer to do community-based oral health work

🞎 not important 🞎 Slightly important 🞎 important 🞎 essential

1. Aware of promoting oral health message importance

🞎 not important 🞎 Slightly important 🞎 important 🞎 essential

1. Demonstrate a health behavior that reflects the health educator’s image

🞎 not important 🞎 Slightly important 🞎 important 🞎 essential

**Rights’ respect domain**

1. Respect beliefs, culture and Decent treatment for various groups of children, teachers, administrators and parents.

🞎 not important 🞎 Slightly important 🞎 important 🞎 essential

1. Treat students equally with no discrimination to ethnicity, belief, gender, or social background

🞎 not important 🞎 Slightly important 🞎 important 🞎 essential

**respecting laws and regulations domain**

1. Adhere to principles, laws and recommendations governing the work of oral health educators to interact with children within elementary schools and to apply oral health education procedures and preventive measures.

🞎 not important 🞎 Slightly important 🞎 important 🞎 essential

1. Aware of the limitations of their work and not to override

🞎 not important 🞎 Slightly important 🞎 important 🞎 essential

Additional suggested Competencies:

________________________________________________________________________________________________________________________________________________________________________________________________________________________________________________________________________________________________________________________________________________________

__________________________________________________________________________________________________________________________________________________________________________________________________________________________________________________________________

**Thank you very much for your valuable participation in answering this questionnaire.**

**Delphi s’ second cycle questionnaire**

**Introduction:**

Dear experts, the researchers would like to thank you again for your participation and cooperation in the first Delphi s’ cycle, and summarize for you what was achieved in the first cycle:

1- the fallowing codes: 1, 2, 3 and 4 were given for your answers corresponding to ‘not important’, ‘Slightly important’, ‘important’, and ‘essential’, respectively

2- The evaluation mean value of ≥2.5 has been considered acceptable for entering the next Delphi’s cycle.

3- All competencies, according to your assessment, exceeded the limit value, as all mean values ​​were >2.5

4- Six additional competencies were suggested by you. We incorporate them into the competency items to be evaluated by you in the 2nd Delphi cycle. competencies in the same way as the first session.

Again, thank you very much for your cooperation by sharing your opinions, which we consider extremely important for our research.

**Expert name:** ______________________ **Email:** ________________________

Please Add ☑ in the box of the corresponding answer which represents your evaluation about each competency to be a core competency for oral health educators in elementary schools in Syria during COVID- 19 pandemic.

**Cognitive(Knowledge)**

**public health domain**

1. Explain briefly the oral manifestations related to common childhood diseases

🞎 not important 🞎 Slightly important 🞎 important 🞎 essential

**Oral and dental diseases domain**

1. Distinguish some oral lesions other than dental caries and periodontal disease

🞎 not important 🞎 Slightly important 🞎 important 🞎 essential

**The prevention of Oral and dental diseases and injuries domain**

1. Explain bad oral habits (nail biting and finger sucking) negative effects on oral health.

🞎 not important 🞎 Slightly important 🞎 important 🞎 essential

**Other cognitive competencies domain**

1. Familiarity with different oral health education methods including online education

🞎 not important 🞎 Slightly important 🞎 important 🞎 essential

**Skills**

**Communication skills domain**

1. Communicate with students with special needs who are integrated into schools

🞎 not important 🞎 Slightly important 🞎 important 🞎 essential

**Organize domain**

1. Think systematically

🞎 not important 🞎 Slightly important 🞎 important 🞎 essential

**Thank you very much for your valuable participation in answering this questionnaire**

**Final statement Delphi cycle questionnaire**

**Introduction:**

Dear experts, thank you very much for your valuable participation, which was extremely important in our research, as we have reached, thanks to you, the final stage, which is the stage of final consensus statement, where you, as experts, are asked to submit a final decision by consensus or non-compliance with the list of competencies that we will put in your hands.

**Expert name: ______________________ Email: ________________________**

**Cognitive(Knowledge)**

**public health domain**

1. Knowledge about the basics principals of public health and its maintenance including Personal protection.
2. Realize latest important developments in public health
3. Familiarity with common diseases prevalent in schools
4. Familiarity with emergency diseases in schools
5. Explain briefly the oral manifestations related to common childhood diseases

**Description of the Anatomical elements of the oral fossa domain**

1. Describe the Anatomical elements of the oral fossa

**Describing and identifying the teeth’s anatomical elements domain**

1. Identify tooth parts
2. Identify tooth layers
3. Distinguish temporary from permanent teeth
4. Distinguish teeth’s shapes
5. Explain the sequence of teeth’s eruption

**Explanation of basic dental functions domain**

1. Explain of basic dental functions

**Oral and dental diseases domain**

1. Define dental caries
2. Identify dental caries’ pathogenesis
3. Identify dental caries’ main symptoms and signs
4. Explain dental caries’ development
5. Define periodontal infections
6. Identify periodontal infections’ pathogenesis
7. Identify periodontal infections’ main symptoms and signs
8. Explain periodontal infections’ development
9. Distinguish some oral lesions other than dental caries and periodontal disease

**The prevention of Oral and dental diseases and injuries domain**

1. Explain the nutrition’s importance in preventing oral diseases
2. Describe the means of oral health preservation
3. Explain the role of oral health means in preventing from tooth decay
4. Explain the role of oral health means in preventing from periodontal infections
5. Explain the using methods for oral health preservation means
6. Explain the fluorides’ role and its various applications in preventing tooth decay
7. Summarize fissures sealant’ s role in preventing tooth decay
8. Explain the harmful effects of smoking in all forms on oral and public health
9. Determine ways to avoid oral and dental injuries at school
10. Briefly explain how to handle oral and dental injuries in school
11. Explain bad oral habits (nail biting and finger sucking) negative effects on oral health.

**Other cognitive competencies domain**

1. Realize the regulatory principles, limitations and responsibilities related to the health educators’ work in elementary schools
2. Distinguish the social, economic, cultural and intellectual characteristics related to target groups
3. Familiarity with different oral health education methods including online education

**Skills**

**Communication skills domain**

1. Communicate with different age groups of pupils in elementary schools
2. Communicate with the work team and administrators
3. Interact with the target group and use body language
4. Motivate the target group to periodically visit the dentist and break the fear barrier.
5. Communicate with students with special needs who are integrated into schools

**Tools investment domain**

1. Effective investment of the demonstration tools
2. Use computer and new technologies for oral health education purpose

**Training domain**

1. Guide and apply fluoride mouth rinse procedures in elementary schools.
2. Train pupils on using of oral health means

**Organize domain**

1. Effectively use oral health education time
2. Report tasks Achievement details
3. Think systematically

**Attitude**

**Personal characteristics domain**

1. Demonstrate responsible, careful and patient behavior while performing their work
2. Demonstrate willingness to volunteer to do community-based oral health work
3. Aware of promoting oral health message importance
4. Demonstrate a health behavior that reflects the health educator’s image

**Rights’ respect domain**

1. Respect beliefs, culture and Decent treatment for various groups of children, teachers, administrators and parents.
2. Treat students equally with no discrimination to ethnicity, belief, gender, or social background

**respecting laws and regulations domain**

1. Adhere to principles, laws and recommendations governing the work of oral health educators to interact with children within elementary schools and to apply oral health education procedures and preventive measures.
2. Aware of the limitations of their work and not to override

🞎 I accept this list to be the final competencies’ list for oral health educators in elementary schools

🞎 I don’t accept this list to be the final competencies’ list for oral health educators in elementary schools

Reason _______________________________________________________________________________________________________________________________________________________________________________________________________________________________________________________________________________________________________________________________________________________________________________________________________________________________
